# Our Book Table

**Published:** 1884-08

**Authors:** 


### OUR BOOK TABLE.

THE ROLLER BANDAGE, by WILLIAM BARTON HOPKINS, M. D., *Surgeon to the out-departments of Pennsylvania, Episcopal and University Hospitals, Assistant Demonstrator of Surgery in the University of Pennsylvania, Fellow of the College of Physicians of Philadelphia, etc.* With seventy-three illustrations. Philadelphia: J. B. Lippincott & Co.

Every one who may be expected in the course of his avocation to apply a bandage, should understand the principles which govern it; but every such person does not, and this book was written for the purpose of giving the necessary information. To obtain the best results from this necessary surgical appliance it should be used according to definite rules, varying with each case, and these are hard to comprehend without an actual demonstration. The author has hit upon the happy idea of obtaining photographs from actual life, showing the various methods of application, and from them engravings have been made, and it is upon these that he mainly relies in teaching. Any one who has seen the roller applied by a master of the art, recognizes the importance of a study of its mysteries, and to those who have not had hospital opportunities, and indeed for many who have, this book will prove invaluable.

HEALTH HINTS FOR TRAVELERS: BY JOHN C. SUNDBERG, M. D. Published by D. G. Brinton, Philadelphia. 1884.

This little work sets forth in plain language and in condensed form very many useful suggestions for travelers. Illness at home is bad enough, but among strangers it is doubly afflictive. Changes of diet and habits are always hazardous, and the tourist, who at home perhaps pays strict attention to hygienic laws, finds that abroad his care for himself is necessarily relaxed. The author of the work under notice is an old voyager, has had experience of all climates, and is therefore quite competent to advise those who are leaving home for their first extended tour. His directions have reference to the comfort of pilgrims as well as their health. It is full of counsel regarding the proper dress and equipment of travelers by sea and by land, by rail, in carriages, and on foot. There are some useful prescriptions for sickness, and directions what to do in case of accident. Altogether, the book is one that every intending tourist should carefully read.

TRANSACTIONS OF THE NEW YORK ODONTOLOGICAL SOCIETY FOR 1883. The S. S. White Dental Manufacturing Company, Philadelphia. 1884.

Perhaps there is none of our local professional societies whose meetings and discussions have attracted the attention of dentists to a greater degree than have those of this Association. Professional men everywhere have considered it an honor to be invited to address the members, and before them have been read some of the ablest papers that have graced our professional literature. Within the membership is comprised some of the most intelligent men of dentistry, and it will therefore readily be conceived that the discussions have been of a high order, and that the Book of Transactions will have a permanent value for every enquiring mind.

The volume for 1883 is issued in a style uniform with the preceding ones, and it is unnecessary for us to say more concerning it. The S. S. White Dental Manufacturing Company is known throughout the world for the excellence of the stock and implements that it makes. It seems very rapidly to be coming to the front as a publisher of professional books.

SIXTEENTH AND SEVENTEENTH ANNUAL REPORTS OF THE PEABODY MUSEUM OF ARCHÆOLOGY AND ETHNOLOGY: Presented to the President and Fellows of Harvard College. Cambridge: Printed by order of the Trustees. 1884.

To any one who has visited the Peabody Museum at Cambridge and met its learned and enthusiastic curator, Prof. F. W. Putnam, any extended notice of the work that is being done there is quite superfluous. To those who have not had the privilege, it would be impossible to convey an idea of the wealth of archæological and ethnological relics that are there accumulated. We have before this taken occasion to express our personal obligations to the curator and his able assistants, and we can now only urge upon thinking, investigating dentists, an examination as careful as circumstances will permit, of the treasures there collected, assuring them that they will be repaid a thousand fold for the time spent.

NOTES UPON HUMAN REMAINS FROM THE CAVES OF COAHUILA, MEXICO, BY CORDELIA A. STUDLEY; Assistant in the Peabody Museum at Cambridge.

This is the record of a most exhaustive study upon some very interesting human relics, procured in 1880 by Dr. Edward Palmer, from four caves in the limestone formation in the State of Coahuila, Mexico. Miss Studley brings to her task a rare intelligence, and a thorough knowledge of ethnological science, and the result is a monograph of permanent scientific value. To any one engaged in the study of dental and cranial development, a reading of this pamphlet will be intensely interesting.

LISTERINE. *An antiseptic for internal and external use. Especially adapted to Dental Practice.*

This is an advertising pamphlet, and it is probable that some of the squeamish purists will take us to task for noticing it in this place. But while Listerine is a proprietary remedy it is far from belonging to the quack school. The formula is printed upon every label, and there is no secret in its manufacture. We believe that a notice of the pamphlet here will tend to more good than would the most elaborate review of a work on the Bacillus of Bunions, and surely that would not be considered foreign to these pages.

The little work is devoted to giving the practical experience of many representative dentists and physicians in the use of Listerine, with some considerations of its therapeutic value. The copy that we have received is beautifully bound, and makes a very handsome volume. We have recommended the article; we now commend the book, and if anyone is harmed by either he may hold us responsible for the damages.

BERICHT UBER DIE VORTRAGE DES HERRN ZAHNARZT, W. HERBST, AUS BREMEN, *gehalten am* 14., 15., 16. *Februar*, 1884, *in der Poliklinik des Herrn* PROF. BUSCH, IN BERLIN.

The lately presented methods of Herr Herbst have attracted the attention of dentists everywhere. We have only a slight practical experience in them, but they are endorsed by good authority, and we believe his theories are worthy the investigation of every progressive dentist. In an early number we expect to be able to present the opinions of one who all will agree is entirely competent to analyze the whole matter. The pamphlet is a reprint from "*Correspondenz-Blatt fur Zahnarzte*" for April, 1884.

We have also received the following pamphlets.

*Select Topics in the Surgery of the Nervous System* and *Report on Surgery;* Read before the Illinois State Medical Society, May, 1884, by Roswell Park, M. D.,; Professor of Surgery in the Medical Department of the University of Buffalo. Reprinted from the *Weekly Medical Review.*

*On Embryology, with special reference to the development of the Teeth and contiguous parts.* By J. L. Williams, D. D. S., New Haven, Conn.

*Address on Practical Medicine,* delivered before the American Medical Association, at the Thirty-fifth Annual Meeting, held in Washington, D. C., May, 1885. By John V. Shoemaker, A. M., M. D.

*The Dense Water of the Ocean.* By Lewis H. Beebe.

*How to Grow Fine Celery.* A new method. By Mrs. H. M. Crider.

*Annual Announcement and Catalogue of the College of Physicians and Surgeons.* Baltimore, Md., 1884.

